# Cytotoxic and anti-proliferative effects of *Rosa beggeriana* Schrenk extracts on human liver and breast cancer cells 

**Published:** 2019

**Authors:** Ozra Zarei, Mohammad Mehdi Yaghoobi

**Affiliations:** 1 *Department of Biotechnology,* * Graduate University of Advanced Technology, Kerman, Iran* *.*; 2 *Department of Biotechnology,* *Institute of Science and High Technology and Environmental Sciences, Graduate University of Advanced Technology Kerman, Iran.*

**Keywords:** Rosa beggeriana Schrenk, Liver cancer cell, Breast cancer cell, Cytotoxicity, Apoptosis

## Abstract

**Objective:**

*Rosa beggeriana *Schrenk has been consumed in Iranian traditional medicine. In contrary to its close species (e.g. *R. canina*), there is no data on its medicinal properties. Therefore, we explored possible cytotoxic effects of *R. beggeriana* against two cancer cell lines.

**Materials and Methods:**

The cytotoxic and anti-proliferative effects of *R. beggeriana* ethanolic and aqueous extracts on human liver cancer cells (LCLPI 11), breast cancer cells (MCF-7) and fibroblast-like cells (HSkMC) were evaluated by MTT, BrdU and TUNEL assays.

**Results:**

Following 48 h, IC_50_ values for LCL-PI11 and MCF-7 cells were found to be 3.9 and 4.2 μg/mL for aqueous extract, and 2.3 and 2.7 μg/mL for ethanolic extract, respectively.

BrdU assay data verified the MTT results and showed that both extracts inhibit cell proliferation as much as 5-fluorouracil does (p<0.05). The ethanolic extract had a more marked inhibitory effect compared to the aqueous extract (p<0.05). Besides both extracts were less effective against HSKMC cells compared to other cells lines.

TUNEL assay results demonstrated that following 48 h, the aqueous extract induced about 19 and 24% apoptotic death in the LCL-PI 11 and MCF-7 cells, respectively (p<0.05). While at the same time, the ethanolic extract was more potent and caused about 83 and 91% death in the LCL-PI 11 and MCF-7 cells, respectively (p<0.05).

**Conclusion:**

These data indicate that both extracts have anti-proliferative and pro-apoptotic activities on these two cancer cell lines and these effects were more pronounced then their activities against normal cells. Also, the ethanolic extract was more potent than the aqueous extract. Further researches are necessary for finding and isolating effective anticancer ingredient of *R. beggeriana*.

## Introduction

According to the world health organization (WHO) statistics on global health, cancer is considered the second cause of death in the world following ischemic heart disease and stroke. Ischemic heart disease and stroke, account for 15.2 million deaths annually. Whereas the number of global mortality due to cancer reached > 9.6 million in 2018 (WHO, GLOBOCAN 2018 ▶). Despite recent advances in medicine, treatment of cancer is a major burden nowadays and exploring new approaches to fight against cancer is an attractive field in biomedical research. The side effects of conventional cancer treatments like chemotherapy and radiation therapy propel researchers to identify new sources of drugs with higher specificity and fewer side effects.

Nowadays, medicinal plants are widely utilized in traditional medicine throughout the world (Atanasov et al., 2015 ▶). Even in modern medicine, they become of great interest owing to their various applications. They offer a vague source of biologically active compounds with potential to prevent, delay, or cure disorders such as cancer. The high incidence of cancers, and the tendency toward traditional medicine as an alternative form of health care, motivate researches to consider antitumor properties of medicinal plants (Newman and Cragg, 2016 ▶; Tariq et al., 2017 ▶).

*Rosa beggeriana* Schrenk (Rosaceae) is natively distributed in many regions of Middle East including Iran. It has been used in Iranian traditional medicine for a long time as an antihypertensive and diuretic agent and for treatment of kidney stones (Amiri and Joharchi, 2013 ▶). *R. beggeriana* is known in Iran as “*wild Nastaran*” and is similar to the domestic “*Nastaran*” (*Rosa canina *L.) which is cultivated for agricultural purposes. The color of petals of *R. beggeriana* is white, whereas *R. canina* has pink petals. There is also a difference in the shape of their fruit. The fruit of *R. beggeriana* is circular whereas *R. canina* fruits are urceolate. Due to high content of ascorbic acid, phenolics and flavonoids, *R. canina* fruits have antioxidant, antimutagenic, anti-inflammatory and anticarcinogenic effects (Deliorman et al., 2007 ▶; Chrubasik et al., 2008 ▶; Lattanzio, et al., 2011 ▶; Roman et al., 2013 ▶). Tumbas and colleagues fractionated vitamin C and polyphenols from *R. canina* and evaluated their effects on HeLa, MCF7 and HT-29 cell lines. They observed that the vitamin C fraction did not inhibit the growth of tested tumor cells. While polyphenols had anti-proliferative activities (Tumbas et al., 2012 ▶). Recently anti-proliferative effects of *R. canina* extracts on human colon cancer cell line was shown (Jimenez et al., 2016 ▶). Jimenez and colleagues reported that all the extracts (total extracts), vitamin C, neutral polyphenols and acidic polyphenols of *R. canina *showed high cytotoxicity after 72 h of incubation. They also observed that rosehip (the accessory fruit of the rose plant) extracts were powerful antioxidants with antiproliferative effect on Caco-2 cells (Jimenez et al., 2016 ▶). *R. beggeriana* species is very close and similar to *R. canina* and is considered its wild counterpart in Iran. Nevertheless, *R. beggeriana* is less explored and amazingly, there is no published data on *R. beggeriana* medicinal properties or its components. Therefore, to explore the medical application of *R. beggeriana*, we considered to evaluate anti-proliferative and cytotoxic effects of aqueous and ethanolic extract of *R. beggeriana *fruit against liver and breast cancer cell lines and fibroblast-like cells.

## Materials and Methods

**Plant materials and extraction**

Plant fruits were collected from Do daran village (29°25'N 57°19'E) located at 30 Km South-West of Rayen city at Kerman province of Iran in September 2010, and authenticated by Dr. Seyed Mansoor Mirtadzadini, plant systematics specialist (Herbarium voucher No. 2125). The fruits were taken when they became fully ripened. The samples were transported on ice bag to laboratory and stored at -80ºC until extraction. For extraction, these materials were rinsed with distilled water and then, finely ground.

**Preparation of the aqueous extract**

Here, 40 g of the plant fruits was homogenized in a mortar by adding liquid nitrogen. Then, 80 ml distilled water was added to them and shaken for 48 h at 4°C, in a light-proof box. The samples were centrifuged at 12,000 × *g* for 10 min at 4°C. The supernatant was lyophilized by a freeze dryer and dissolved at desired concentrations. Finally, the extract solution was sterilized by 0.22 micron syringe filter and stored at -20°C freezer.

**Preparation of the ethanolic extract**

Here, 150 g of plant fruits was homogenized in a mortar by adding liquid nitrogen. Subsequently, 400 ml ethanol was added to them and shaken for 48 h at 4°C, in the dark. Then, the solution was filtered through a Whatman No. 1 filter paper by vacuum pump. Rotary evaporation was used to evaporate the solvent/water and the remaining solvent/water was air-dried. The extract solution was sterilized by 0.22 micron syringe filter and stored at -20°C freezer.

Ethanolic extract (R.B.E) and aqueous extract (R.B.A) were dissolved in DMSO and culture media respectively to make a 400 µg/mL stock solution. 

**Cell culture and assessment of cell viability**

Human liver cancer cell line (LCL-PI 11, briefly LCL), breast adenocarcinoma (MCF-7) and human fibroblast-like skeletal muscle cell (HSkMC, briefly SKM), were obtained from the National Cell Bank of the Pasteur Institute of Iran (Tehran, Iran). To compare the effects of the two extracts, normal HSkMC cells were used in all trials along with the two other cancer cells. The cells were grown in RPMI 1640 medium supplemented with 10% FBS (Invitrogen, USA) 100 IU/mL penicillin, 100 μg/mL streptomycin and 50 ng/mL amphotericin B, in an incubator at 37°C with 5% CO_2_. The cell viability was determined by 0.4% trypan blue (Sigma, USA) staining, according to the manufacturer’s instructions. 

**Cytotoxicity assay**

The cytotoxicity of the two extracts was measured by Cell Proliferation Kit (MTT) (Roche Applied Science, Germany) according to the manufacturer’s instructions. Briefly, 5000 cells/well of LCL, MCF-7 and SKM cells were separately seeded in 96-well plates in quadruplicate rows and fed with 100 µL culture medium. On the next day, the cells were treated with eight concentrations of the extracts (0, 0.3, 0.6, 1.2, 1.8, 2.4, 3.0, 3.6, 4.2 μg/mL of R.B.A and 0, 0.2, 0.4, 0.8, 1.2, 1.6, 2.0, 2.4, 2.8 μg/mL of R.B.E) for 48 h. 5-Fluorouracil (5-FU) was also used as a positive control drug at 20 or 4.6 μM (equivalent to IC_50_ value) against the LCL and MCF-7 cells, respectively. After 48 h, MTT labeling reagent was added to the medium for 4 h. The solubilization solution (10% SDS in 0.01 M HCl) was then added to each well and the plates were kept at the same condition, overnight. Absorbance (A) of the samples was read on Biotek ELISA reader (Cedex, France) at 490 and 680 nm. To correct background noise, the 680 nm OD background has been subtracted from the 490 nm OD total signal. The viability percentage was determined by the following formula: 

Cell viability (%) = (A of treated cells/A of control cells) ×100. 

Then, mean of four experiments (4 wells/plate) was considered for analysis. IC_50_ value was calculated by ED50 plus V1.0 software.

**Cell proliferation assay**

The effect of the extracts on cell proliferation and DNA synthesis was monitored by Cell Proliferation ELISA, Bromodeoxyuridine (BrdU) Kit) Roche Applied Science, Germany) as recommended by the manufacturer. Firstly, LCL, MCF-7 and SKM cells were seeded in 96-well plates and treated with the same concentrations as those used in the MTT assay, for 48 h. Then, BrdU labeling solution was added to the wells for 3 h. After removing labeling medium, FixDenat was added for 30 min at 20°C and was then removed thoroughly. At this time, the samples were incubated with anti-BrdU-POD working solution for 90 min at 20°C. Next, after three times of washing with PBS, the wells were incubated with substrate solution for 20 min at 20°C. Finally, absorbance of the wells at 370 nm (and 490 nm as reference wavelength) was quantified using an ELISA reader. Absolute absorbance was calculated as A_370_-A_490_. All experiments were performed at least in three wells/plate.

***In situ*** **cell death detection**

Terminal deoxynucleotidyl transferase (TdT) mediated-16-deoxyuridine triphosphate (dUTP) nick end labelling (TUNEL) technique was used for detection of apoptotic effects of the extracts. In this experiment, In Situ Cell Death Detection Kit, TMR Red (Roche Applied Science, Germany) was used. LCL and MCF-7 cells were grown on cover slip and treated with 2.4 and 1.6 μg/mL R.B.A and R.B.E, respectively for 48 h. Treated cells were then rinsed with PBS twice, dried in air and fixed with freshly prepared 4% paraformaldehyde for 1 h at room temperature. Afterwards, they were incubated in permeabilization solution (0.1% Triton X-100 in 0.1% sodium citrate) for 2 min on ice and rinsed twice with PBS. Then, the TUNEL reaction mixture was added and incubated for 60 min at 37°C in a humidified atmosphere in the dark. DNase I was added at room temperature for 10 min to positive control samples and the labeling solution (i.e. nucleotide mixture without terminal transferase) was added to negative control slides. Finally, the cells were observed under a fluorescence microscope (Axioplan 2, Zeiss, Germany) at 590 nm. The percentage of apoptosis was calculated as number of TUNEL-positive cells/number of total cells×100 in five different fields and the average of them was considered. 

**Statistical analysis**

All data were expressed as mean ± standard deviation (S.D) of at least three replicates. Statistical data were analyzed by One-way ANOVA followed by Duncan test in the SAS software. IC_50_ for all of extracts was determined by ED50 Plus V1.0 software. A *P *value* <*0.05 was considered statistically significant.

## Results

**Assessment of cytotoxicity using MTT assay**

**Figure 1:**
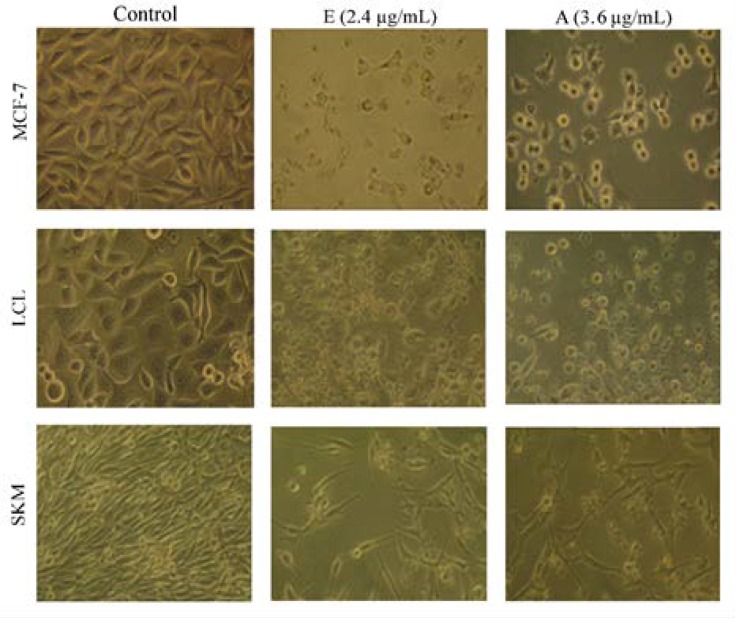
Morphological changes of MCF-7, LCL and SKM cells following 48 h treatment with one of the higher concentration of ethanolic (i.e. E 2.4 µg/mL) or aqueous extract (i.e. A 3.6 µg/mL) of *R. beggeriana*. Massive cell death and cell debris are evidently seen in the treated MCF-7 and LCL cells. The left column shows cells in control dishes, which were cultured in complete medium without any treatment. Magnification 320 X

Following treatment of the MCF-7, LCL and SKM cells with aqueous and ethanolic extracts for 48 h, the morphology of all of the cells altered clearly especially at higher concentrations (Figure 1). The detached round cells which were floating in the medium with wrinkled nucleolus, bubbled membrane as well as cell debris, were obviously seen in MCF-7 and LCL cells. Such morphological features, which are signs of cell death, were rarely observed in SKM cells, and were not seen in untreated cells or in those treated with DMSO (Figure 1).

The IC_50 _values for LCL cells were 3.9 and 2.3 µg/mL for aqueous and ethanolic extracts, respectively (Table 1). It means that the ethanolic extract was stronger than the aqueous extract. The obtained IC_50_ values for MCF-7 cells were 4.2 and 2.7 µg/mL for aqueous and ethanolic extracts, respectively (Table 1). 

As the concentration of extracts increased, the viability of the cells decreased. Similar to what observed for LCL cells, the ethanolic extract was more potent than the aqueous extract against MCF-7 cells. The results also demonstrated that the effect of DMSO, as solvent, in ethanolic extract was negligible. Therefore, the features observed following applying ethanolic extract are attributable to the extract per se.

**Table 1:** IC_50_ values for aqueous and ethanolic extracts for the three treated cells, as determined by MTT assay

Cells	IC_50_ (aqueous) µg/mL	IC_50_ (ethanolic) µg/mL
MCF-7	4.2	2.7
LCL	3.9	2.3
SKM	4.6	3.6

**Figure 2:**
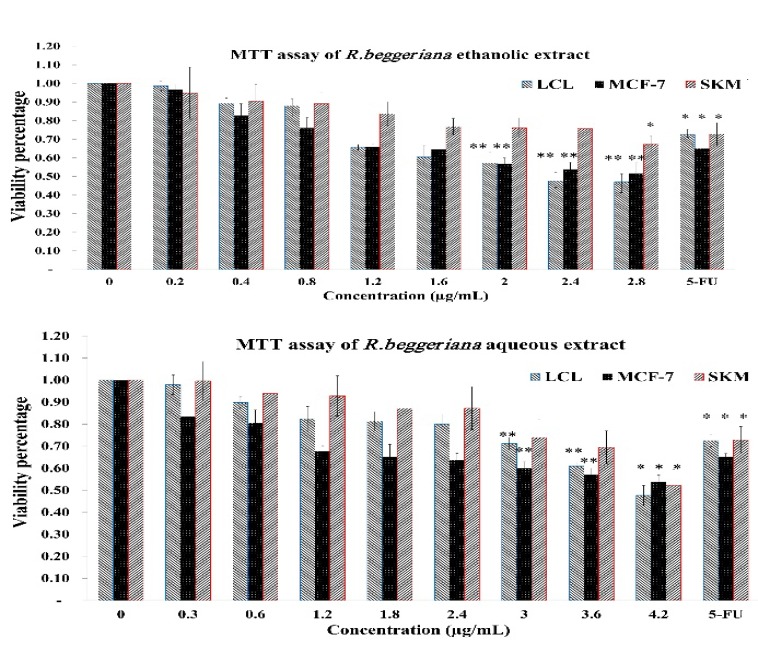
MTT assay done to investigate cytotoxic effects of *R. beggeriana* extracts on LCL, MCF-7 and SKM cells. The cells were incubated with eight concentrations of ethanolic and aqueous extracts and 5-fluorouracil for 48 h. Data are presented as the mean±SD of independent quadruplicate experiments. ** indicate p<0.01, and *indicate p<0.05 vs. control which is labelled as zero concentration

Statistical analysis revealed that among the eight concentrations applied, the three higher concentrations (i.e. ≥3 µg/mL for aqueous extract and ≥2 µg/mL for ethanolic extract) against MCF-7 and LCL cells were significantly cytotoxic in comparison to the untreated control group (p<0.05 for both cases). Whereas only the highest concentration (4.2 and 2.8 µg/mL for aqueous and ethanolic extracts, respectively) induced significant toxicity in SKM cells (p<0.05) (Figure 2). The cytotoxicity of 5-FU in all three cell lines was also significant compared to two extract (p<0.05).

**Evaluation of cell proliferation by BrdU assay**

In the next step, to assess effect of the extracts on cell proliferation, DNA synthesis was quantified by BrdU assay. The same eight concentrations of aqueous and ethanolic extracts that were used in the MTT assay, were also applied in this step on all three cell lines.

**Figure 3:**
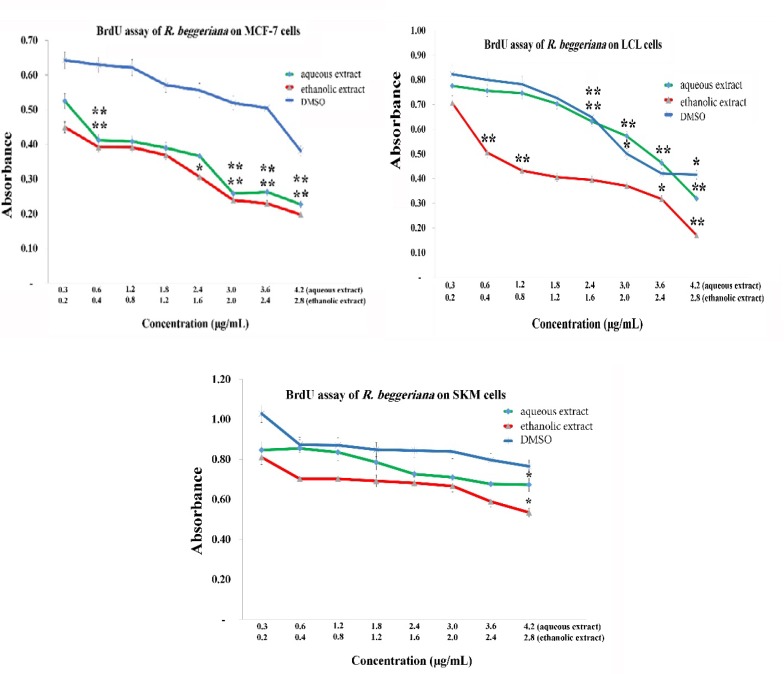
Dose-response curve of the effects of eight concentrations of *R. beggeriana* extracts on MCF-7, LCL and HSkMC cells proliferation following 48-h treatment. The data are expressed as optical density of bromodeoxyuridine incorporation. ** indicate p<0.01, and *indicate p<0.05 vs. control

The results of BrdU assay demonstrated that both extracts had inhibitory effect on DNA synthesis and cell proliferation. Interestingly, ethanolic extract of *R. beggeriana *had greater inhibitory influence than aqueous extract on proliferation of all three cell lines (p<0.05), (Figure 3). It lessened proliferation of all three cell lines in a dose-dependent manner. MCF-7 and LCL cell lines were more markedly affected by either aqueous or ethanolic extracts compared to SKM cells (p<0.05). In other words, the SKM cell line was more resistant to the inhibitory effects of the extracts and its proliferation was partly suppressed by the highest concentration of the extracts. These findings were similar to the MTT assay results. The three lower concentrations of ethanolic extract, reduced proliferation of LCL cells sharply. However, the result of ethanolic extract on proliferation of MCF-7 was near to that observed for the aqueous extract. In addition, the inhibitory effect of DMSO on cell proliferation was less pronounced compared to the extracts (Figure 3).

**Apoptosis detection using TUNEL test**

In the last step, the TUNEL test was used to evaluate the type of cell death in the two cancer cell lines, MCF-7 and LCL. The obtained data indicated that in LCL cells, 19 and 90% apoptosis rates were observed following treatment with aqueous and ethanolic extracts, respectively. 

**Figure 4:**
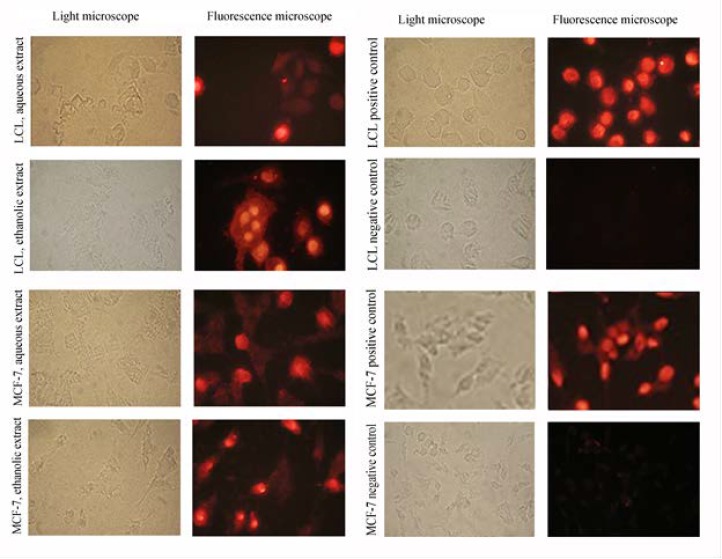
Analysis of *R. beggeriana* extracts-induced apoptosis in LCL and MCF-7 cells by TUNEL test: right column shows the stained cells under fluorescence microscope. Left column shows the same field under light microscope. The positive control (cells treated with DNase I) and negative control (cells that were not incubated with terminal transferase) are also shown

Whereas, in MCF-7 cells 24 and 83% apoptosis rates were observed following treatment with the aqueous and ethanolic extracts, respectively (Figure 4). In accordance with MTT and BrdU assay results, the TUNEL data also confirmed that ethanolic extract is significantly more powerful than aqueous extract and induced higher levels of apoptosis (p<0.01). 

## Discussion

Nowadays, it is important to explore new sources of natural medicines because the demand for such drugs is increasing. Plants, especially those that are traditionally consumed as nutrient or food additive, are an unexplored source for searching new medicines. Aerial parts of *R. beggeriana *including rosehips are traditionally used as food with health and nutritional benefits in many regions including Iran and it is probably a suitable source of natural antioxidants.

We investigated the cytotoxic and antiproliferative effects of two aqueous and ethanolic extracts of *R. beggeriana *against two cancer cell lines, MCF-7 and LCL, as well as normal SKM cells. Our findings revealed that both extracts were toxic. The survival rate of MCF-7 and LCL cells was strongly decreased as the concentration of the extracts increased. Nonetheless, the survival of SKM cells was slightly reduced with a mild slope. The survival of the cells fell rapidly when the ethanolic extract was applied. The obtained IC_50 _showed that LCL cells were the most sensitive among all to both aqueous and ethanolic extracts, whereas the SKM cells were affected less than the others. The IC_50 _of DMSO showed that the presence of DMSO has no significant toxic effect on the cells (data not shown). Thus, the observed toxicity can be surely attributed to the ingredients in the ethanolic extract.

Evaluation of cell proliferation by BrdU assay, also demonstrated that DNA synthesis was decreased in all of the cells upon treatment with both extracts in a dose-dependent manner, but with different slopes. This assay also confirmed that ethanolic extract was more effective than aqueous extract. The aqueous extract had an outstanding inhibitory effect on proliferation of MCF-7 cells which was comparable to the ethanolic extract. 

 Interestingly, the effect of both extracts was near to that of 5-FU (a routine drug in cancer chemotherapy) and even at some concentrations, was more marked than 5-FU effect. This finding suggests that *R. beggeriana* is an appropriate candidate to be screened for new medicines. Our findings showed an IC_50_ for 5-FU that was slightly higher than the previously reported one (Rajkapoor et al., 2007 ▶). This can be due to emergence of partial resistance to 5-FU or shorter treatment period (i.e. 48 h) in our experiments. 

Altogether, the results of MTT, BrdU and TUNEL assays confirmed that ethanolic extract was more effective than aqueous extract. As plants are rich in a great variety of bioactive compounds especially secondary metabolites, there is no single solvent that solubilize all of them. The composition of materials obtained by organic solvent extraction is more complex containing non- to moderately-polar small molecules. Whereas a water-soluble extract contains polar small- to medium-sized molecules (McCloud, 2010 ▶). Some parameter in extraction process, such as time and temperature, can also affect the type of compounds that are extracted. Thus, the noticeable difference between the aqueous and ethanolic extracts property is due to differences in their composition. Further chemical analysis of the extracts can reveal their components. 

Nadpal et al. found that quercetin, gallic acid and protocatechuic acid are dominant compounds in *R. canina* rose hip (Nadpal et al., 2016 ▶). They also observed cytotoxic activity of *R. canina* against the HeLa cell line among several others (HeLa, MCF7, HT-29 and MRC-5) (Nadpal et al., 2016 ▶). 

Roman et al. reported that antioxidant activity of *R. canina* has a good correlation only with ascorbic acid content and total polyphenols (Roman et al., 2013 ▶). 

Quality and quantity of compositions of *R. canina *also significantly differ even among cultivars, regions and altitudes. Jimenez and colleagues recently reported that ascorbic acid and neutral phenols content of *R. canina* vary quantitatively among different geographical zones. In addition, among four species of *Rosa*, *R. canina* has the highest levels of ascorbic acid and antioxidant activity (Jimenez et al., 2017 ▶).

According to the previous studies, *R. canina* is a rich source of ascorbic acid and natural polyphenols, which are both well-known for prevention and treatment of cancer. Although *R. beggeriana* components have not been explored yet, we can suppose that the observed cytotoxic effects of *R. beggeriana* are due to the presence of antioxidants and polyphenols in its extract. Biochemical analysis of *R. beggeriana* will reveal the chemical profile of its components. Ascorbic acid is an antioxidant that scavenges free radicals and oxidants and is believed to lead to cancer prevention. Although it has a protective role, at higher concentrations it is toxic to cancer cells (Falahi et al., 2014 ▶; Jimenez et al., 2016 ▶). It is believed that polyphenols also have anticancer properties. The suggested mechanisms include antioxidant, anti-inflammation as well as modulation of several molecular pathways involved in carcinogenesis. It has been reported that natural polyphenols can arrest cell cycle, induce apoptosis and inhibit angiogenesis and invasion (Zhou et al., 2016 ▶). 

Quercetin is a well-known anticancer compound found in *R. canina* (Fujii and Saito, 2009 ▶). Some of our observations can be attributed to the presence of quercetin. Recently, it has been shown that quercetin inhibits proliferation and induces apoptosis in both hepatocellular carcinoma and breast cancer cells (Duo et al., 2012 ▶; Dai et al., 2015 ▶). Our findings that *R. beggeriana *extracts induce apoptosis in liver cancer cells and breast cancer cells, are similar to the previous reports on *R. canina* extract. Plausibly, quercetin is the major apoptosis-inducer agent in *R. beggeriana *fruit extract.

Indeed, the anticancer effects of polyphenols varied with doses, cancer types and cell lines. The anticancer activities of *R. beggeriana *polyphenols need to be evaluated and their mechanisms of action need further study. It is clear that metabolites derived from plants may target a specific protein in a pathway, or may act synergistically. The vast majority of cancers are dependent on multiple signaling pathway redundancies rather than a single target oncogene. Natural biomolecules in medicinal plants such as *R. beggeriana *typically target multiple signaling pathways.

Collectively, our data demonstrated that *R. beggeriana *have cytotoxic, anti-proliferative and pro-apoptotic activities against human liver and breast cancer cell lines. Both cancer cell lines were found to be more sensitive than the fibroblast-like cells. Further studies are necessary for biochemical characterization and *in vivo* evaluations to identify effective anticancer ingredients of *R. beggeriana*.
